# Investigation and Management of Adnexal Masses in Pregnancy

**DOI:** 10.1155/2016/3012802

**Published:** 2016-03-28

**Authors:** João Cavaco-Gomes, Cátia Jorge Moreira, Anabela Rocha, Raquel Mota, Vera Paiva, Antónia Costa

**Affiliations:** ^1^Department of Obstetrics and Gynecology, São João Hospital Centre, 4200-319 Porto, Portugal; ^2^Porto Medical Faculty, 4200-319 Porto, Portugal

## Abstract

Adnexal masses can be found in 0.19 to 8.8% of all pregnancies. Most masses are functional and asymptomatic and up to 70% resolve spontaneously in the second trimester. The main predictors of persistence are the size (>5 cm) and the imagiological morphocomplexity. Those that persist carry a low risk of malignancy (0 to 10%). Most malignant masses are diagnosed at early stages and more than 50% are borderline epithelial neoplasms. Ultrasound is the preferred method to stratify the risk of complications and malignancy, allowing medical approach planning. Pregnancy and some gestational disorders may modify the levels of tumor markers, whereby their interpretation during pregnancy should be cautious. Large masses are at increased risk of torsion, rupture, and dystocia. When surgery is indicated, laparoscopy is a safe technique and should ideally be carried out in the second trimester of pregnancy.

## 1. Introduction

Adnexal masses originate in the ovaries, fallopian tubes, or surrounding tissue. They may present as different histological types and their clinical significance may vary largely depending mainly on tumor size, clinical presentation, and risk of malignancy. Adnexal masses can be found in women of any age, and pregnancy is not only no exception, but also a period associated with some particular adnexal formations. The systematic use of ultrasound in the first trimester of pregnancy led to increased identification of asymptomatic and once clinically undetectable adnexal masses, bringing to debate their guidance and their implications during pregnancy.

## 2. Materials and Methods

The aim of this study was to review evidence about epidemiology, clinical presentation, medical approach, and management of adnexal masses in pregnancy.

In order to access evidence about epidemiology, natural history, and outcomes, the PubMed Central database was searched to identify studies reporting cases of adnexal masses in pregnancy. The search was conducted using two sets of keywords: (1) “adnexal mass” and “pregnancy”; (2) “ovarian mass” and “pregnancy”. The database was searched without restriction on date of publication. Case reports were not considered. Additional relevant articles were garnered from the references of included papers. Eligibility for inclusion was limited to papers written in English and French. Each abstract obtained through the electronic database was evaluated for relevance, and the full text of each relevant abstract was obtained and evaluated for inclusion. From 137 abstracts initially selected, 72 full-text articles were reviewed for eligibility and 37 were finally included in this review: 29 retrospective studies, 5 prospective studies, and 3 review articles.

## 3. Epidemiology

The prevalence of adnexal masses in pregnancy is estimated to be around 0.19–8.8% [1]. Most cases are diagnosed in the first trimester, and the incidence appears to gradually decrease with increasing gestational age (Table 1) [2–6].

## 4. Clinical Presentation

Adnexal masses in pregnancy are mostly asymptomatic, being diagnosed as ultrasound or surgical findings. During C-sections adnexal masses are found in about 0.3% of the cases [7, 8]. Only a minor proportion of women have symptoms, most frequently pain (Table 2). In about 31 to 72% of cases there will be a spontaneous resolution of the mass (Table 2). The most important predictors for persistence are imagiological morphocomplexity and dimension larger than 5 cm [9]. Masses that persist throughout pregnancy tend to be displaced upwardly out of the pelvic cavity, as the uterus increases in size. Occasionally, they may be incarcerated in the Douglas pouch throughout pregnancy, with subsequent compression of adjacent structures, namely, urinary tract and lower digestive tract, with subsequent symptoms from these organs. The incarceration of the mass within the pelvis can also be responsible for labor dystocia through obstruction the descent of the presenting fetal part.

## 5. Histology (Table 3)

Most adnexal masses in pregnancy are benign and mainly represented by functional cysts (follicular, corpus luteum, and theca lutein cysts), teratomas, and cystadenomas. Nevertheless, malignant adnexal tumors should also be considered. The risk of malignancy is estimated around 0–10% [3, 7, 9–16]. Main series refer that borderline ovarian tumors account for over 50% of the malignant tumors [4, 9, 14, 16, 17].

## 6. Evaluation

Once an adnexal mass is diagnosed, regardless of the associated symptoms (excepting cases of acute abdomen), clinical approach should involve assessment of malignancy risk as well as predictors of tumor persistence and complications. Imaging study (ultrasound with doppler fluxometry and magnetic resonance imaging) associated with serum tumor markers plays a key role in this setting.

### 6.1. Ultrasound

Usually adnexal masses in pregnancy are imagiological findings, but when detected by palpation on physical exam, ultrasound should be the first step to characterize the lesion. Transabdominal and/or transvaginal ultrasound allows morphodimensional evaluation and assessment of mass vascularization and growth, as well as observation of the contralateral ovary and detection of other malignant signs, such as the presence of ascites and peritoneal carcinomatosis [18, 33]. Several ultrasonographic features were associated, in nonpregnant women, with an increased risk of malignancy, such as irregular solid tumor, multilocular and irregular masses with more than 10 cm, septa thicker than 2-3 mm, presence of more than three papillary projections, exuberant blood flow, and presence of ascites or peritoneal masses [34, 35]. Application of IOTA (International Ovarian Tumor Analysis) group rules in ultrasound evaluation of premenopausal and postmenopausal women has demonstrated high sensitivity and specificity at stratifying the malignancy risk [36, 37]. Although there are no studies demonstrating the performance of these models in pregnancy, the previously listed malignant sonographic features seem to be also valid in pregnancy [5, 9, 10, 28, 29].

Nevertheless, ultrasound evaluation of adnexal masses in pregnant women has some limitations. Increased uterine volume makes evaluation difficult after 20 weeks of gestation. Furthermore, fluxometric parameters may be difficult to appreciate in pregnancy, both by the increased flow velocity and lower vascular resistance found at pregnancy and by the significant overlap in fluxometric pattern between benign and malignant masses, with an estimated false positive rate of 48-49% [38, 39]. In addition, germ cell tumors, one of the most common histological types, have less defined malignant sonographic features.

### 6.2. Magnetic Resonance Imaging and Computed Tomography

Magnetic resonance imaging (MRI) should be reserved for those cases in which ultrasound evaluation is inconclusive or insufficient for clinical decision planning [40]. MRI is particularly useful for characterizing large masses, evaluating their relationship with adjacent structures (namely, adjacent organ invasion and compression), and differentiating masses appearing to have adnexal location on ultrasound but actually having a different organ filiation [33]. MRI is also useful, when an advanced stage malignant tumor is suspected, requiring the assessment of the retroperitoneum, abdominopelvic cavity, and lymph nodes. MRI appears to be safe in pregnancy, but caution is advised for its use in first trimester, as well as for the use of gadolinium contrast, because of the potential fetal risks [41].

Despite its better performance at retroperitoneal and lymph node evaluation, Computed Tomography (CT) plays a limited role in pregnancy, because of the potential risk associated with fetal irradiation [32]. Nevertheless, it may be useful for thoracic assessment in case of malignant lesions, provided that abdominal radiation protection is used.

### 6.3. Tumor Markers

The interpretation of tumor markers serum levels in pregnant women requires caution, since they may be physiologically increased during certain stages of pregnancy (Table 4). This is particularly true regarding some oncofetal antigens (alpha-fetoprotein, human chorionic gonadotropin, and CA125), which are involved in biological functions associated with development, differentiation, and fetal maturation [21–24]. Moreover, placental and fetal anomalies (such as preeclampsia, 21 trisomy, and neural tube defects) may also present with an abnormal increase of these markers [21, 25]. The main interest of tumor markers seems to be the differentiation between primary and secondary adnexal tumors and the possibility to monitor their levels to evaluate the therapeutic response and the risk of relapse [32].

## 7. Complications (Table 5)

Most adnexal masses in pregnancy are benign and physiological. Spontaneous resolution is the rule. However, the risk of malignancy is not negligible and other complications may arise such as torsion, rupture, and bleeding. On the other hand, the associated obstetric risk, particularly the risk of miscarriage, preterm labor, and mechanical dystocia, should be also kept in mind. Table 5 summarizes the frequency of the most frequently described complications.

### 7.1. Adnexal Torsion

Adnexal torsion is one of the most frequent gynecological surgical emergencies and about 10 to 20% occur in pregnancy [42–44]. It is defined as complete or partial rotation of the ovarian vascular pedicle on its long axis, usually involving the tube and ovary. If torsion persists over 36 to 48 hours, it is considered a surgical emergency due to the risk of irreversible damage to the functional ovarian tissue [45–47].

Different factors have been associated with an increased risk of adnexal torsion in pregnancy, including gestational age (58% of cases in the first trimester) [2], mass size (greater risk for masses with diameters between 6 and 10 cm) [2, 15], and ovarian stimulation (73% of cases associated with the use of assisted reproductive technology) [48].

Clinical presentation is not specific and usually includes abdominal pain, nausea, vomiting, and eventually fever and leukocytosis [48]. Diagnosis requires a high index of suspicion, but some imaging signs are suggestive, such as larger and more rounded ovary, heterogeneous stromal echogenicity, multiple small size follicles in periphery, decreased intraparenchymal blood flow, and the “Whirlpool sign.”

Treatment of this complication requires surgery. After detorsion of the adnexa, restoration of blood flow should be assessed: if recoloring and decreasing edema are noted, one should only proceed to the aspiration of the hemorrhagic content. Bivalving technique is also described for vascular decompression and restoration of ovarian blood flow [49]. In case of persisting signs of ischemia, adnexectomy is mandatory.

The risk of rupture (0–5%) [11, 16] and other mechanical complications such as hemorrhage and obstruction (9%) [4] is less frequently described.

### 7.2. Malignancy

The occurrence of cancer in pregnancy is an uncommon event, with an overall estimated incidence of 0.02 to 1% [50, 51]. Although rare, ovarian cancer is the second most common gynecological cancer in pregnancy with an incidence of 1 : 12.500 to 1 : 25,000 pregnancies [52].

The proportion of adnexal masses in pregnancy which correspond to malignant tumors varies considerably among published series (0 to 10%, Table 3). It has been estimated that on average 1 out of every 16,000 pregnancies is complicated by an adnexal malignant tumor [53].

Besides the previously mentioned sonographic features suggestive of malignancy, in pregnancy, the lesion size (different cut-offs have been proposed, Table 6) and a growth rate greater than 0.35 cm/week (10 times greater risk of malignancy) are also of note [15].

At younger age groups the most common malignant tumors of the ovary are germ cell tumors and epithelial tumors, and in pregnancy the latter may be even more common than the former (Table 3) [4]. Contrarily to the nonpregnant women, the majority of ovarian tumors are diagnosed in the early stages (80% in stage 1 of FIGO), with a large proportion of those corresponding to borderline tumors [2, 4, 15].

### 7.3. Obstetric Outcome

Depending on their size and location, adnexal masses may be associated with a poor obstetric outcome caused by a mechanical effect. They have been linked to an increased risk of miscarriage, preterm delivery, and dystocia. In addition, prenatal surgical approach to these lesions, either at emergency situations (e.g., ovarian torsion) or on a scheduled basis, is also associated with a significant obstetrical risk (Table 7).

## 8. Treatment

### 8.1. Expectant Management

As mentioned above, most identified adnexal masses in pregnancy resolve spontaneously, requiring no intervention. Therefore, expectant management towards an adnexal cyst in the first trimester should be encouraged, as long as there is a low index of suspicion for malignancy or complications (such as torsion or rupture). In case of persistence, the risk of complications such as acute abdomen, dystocia, or malignancy must be weighed against possible risks of an antepartum surgery. Given a sonographic low risk of malignancy without predictors of complications, clinical and imaging surveillance until postpartum period must be considered and has been supported by the existing retrospective studies [10, 17, 18].

### 8.2. Surgery

Surgical treatment is indicated in cases of acute abdomen, severe clinical manifestations (e.g., hydronephrosis), strong suspicion of malignancy, or anticipated risk of dystocia. Surgical intervention, if not urgent, should in general be delayed until the second trimester because (1) it avoids the period of greatest risk of drug-induced teratogenicity; (2) spontaneous fetal losses due to intrinsic fetal abnormalities have already occurred; (3) the function of corpus luteum has been replaced by the placenta; (4) most functional cysts have disappeared; (5) an acceptable operative field is still available, allowing minimal uterine manipulation and low risk of obstetric complications. A later surgery in pregnancy, at the end of second trimester or at third trimester, may be technically more difficult and result in an adverse obstetric outcome. When indicated for other reasons, cesarean section may also be an opportunity for the surgical management of adnexal masses.

The type of surgical approach, laparotomy, or laparoscopy, has also been subject of debate in the literature. The advantages of laparoscopy are largely the same as for nonpregnant women and include lower operative pain; minor bleeding; early ambulation, reducing the risk of thromboembolic events; early resumption of bowel transit; shorter hospital stay; faster return to activities of daily living; lower risk of surgical wound infection; and less uterine manipulation, with a consequently theoretical lower risk of preterm delivery.

However, some concerns about a laparoscopic approach in pregnancy have been raised: high intra-abdominal pressure may compromise placental perfusion; maternal conversion of carbon dioxide into carbonic acid can result in fetal acidosis; pregnant uterus may be injured during insertion of Veress needle or trocars [54].

Although there are no prospective studies assessing the safety of laparoscopy in pregnancy, main published series show that it is technically feasible and as safe as laparotomy, with a conversion to laparotomy rate lower than 10% [13, 16, 55–59]. The Society of American Gastrointestinal and Endoscopic Surgeons (SAGES) stated that laparoscopy can be safely performed in any trimester of pregnancy [60]. This society has developed some technical recommendations for performing laparoscopy in pregnant women, which include the following: (1) patient must be positioned in left lateral position (to minimize inferior vena cava compression); (2) initial access to the abdominal cavity can be safely performed by open surgery (Hasson) with a Veress needle or by optical trocar, as long as the location takes into account the uterine fundal height and previous incisions; (3) CO2 insufflation with 10–15 mmHg pressure is safe, but intraoperative CO2 monitoring by capnography is recommended; (4) pneumatic compression devices are recommended in intra- and postoperative period as well as early postoperative ambulation (for venous thromboembolism prophylaxis); (5) tocolytics should not be used prophylactically but should be considered perioperatively in the presence of preterm labor signs [60].

When malignancy is suspected, assertive information must be offered to the pregnant woman. Only after precise and clear discussion of all the viable options (not neglecting the risks and benefits towards the obstetrics and oncological outcomes) can the medical approach be guided by the patient decision. Clinical management can vary widely and many factors must be taken into account, namely, (1) gestational age (previability, viability far from term and near term); (2) oncological disease status (suspected malignancy and with disease confined to the ovary, extraovarian dissemination with carcinomatosis but without intraorgan spread or intraparenchymatous organ spread). These factors will interfere with the treatment options (such as complete surgical staging, cytoreductive surgery followed by adjuvant chemotherapy, and neoadjuvant chemotherapy followed by surgery), with relevant impact on neonatal and maternal morbimortality. The surgery is usually performed by laparotomy, but laparoscopic approach may be considered as long as complete staging is respected and all the standard precautions to avoid spread of malignant cells during the procedure are taken (in particular, avoiding tumor rupture during handling of the specimen) [61–63]. The discussion of specific surgical and medical management of ovarian malignancy in pregnancy is beyond the scope of this review.

## 9. Conclusions

Most adnexal masses, which arise in pregnancy, are functional and asymptomatic and resolve spontaneously. Persistent adnexal masses carry a low risk of malignancy, and ultrasonography is the preferred method to assess this risk. In asymptomatic pregnant women without predictors of complications and sonographic findings suggestive of malignancy, expectant management is generally the most suitable option. On the other hand when strong clinical suspicion of malignancy or presence of acute abdomen or severe clinical manifestations occur, surgical treatment is indicated by laparotomy or laparoscopy. Laparoscopy was shown to be a safe and technically feasible approach, with the advantages of a minimally invasive surgery.

## Figures and Tables

**Table 1:** Proportion of adnexal masses detected throughout pregnancy [2–6].

Period	Proportion of cases diagnosed
1st trimester	21.4–75.7%
2nd trimester	10.9–44.4%
3rd trimester	4–22.2%
Postpartum	0–7.1%

**Table 2:** Clinical presentation and frequency of spontaneous resolution of adnexal masses in pregnancy [3, 4, 9–11, 18–20].

	*N*	Asymptomatic	Symptomatic	Spontaneous resolution
Bernhard et al., 1999 [9] (>5 cm/complex)	102	78.4%	21.6% (pain or palpable mass)	68.6%
Zanetta et al., 2003 [18]	79	86.1%	13.9% (pain)	51%
Agarwal et al., 2003 [19]	14	35.6%	50% (pain or discomfort) 14.2% (larger than expected uterus)	NA
Condous et al., 2004 [20]	161	43.7%	56.3% (pain or genital bleeding)	71.7%
Schmeler et al., 2005 [10] (>5 cm)	59	92%	8% (pain)	1,7%
Kumari et al., 2006 [11]	20	50%	35% (pain) 15% (NE)	NA
Balci et al., 2008 [3]	36	30.6%	69,4% (pain)	NA
Aggarwal and Kehoe, 2011 [4] (review)^*⨀*^	809	65.4%	16.8% (pain) 9% (bleeding/dystocia/rupture)	30.7%

NE: not specified; NA: not applicable to study design. ^*⨀*^Review of studies including only surgically managed cases of both benign and malignant tumors (includes the other studies quoted here, except Agarwal et al. (2003) [19] and Kumari et al. (2006) [11]).

**Table 3:** Most frequent histological types of adnexal masses in pregnancy.

	Bernhard et al., 1999 [9]	Soriano et al., 1999 [13]	Whitecar et al., 1999 [14]	Yuen et al., 2004 [12]	Schmeler et al., 2005 [10]	Leiserowitz et al., 2006 [17]	Kumari et al., 2006 [11]	Balci et al., 2008 [3]	Yen et al., 2009 [15]	Ulker et al., 2010 [7]	Aggarwal and Kehoe, 2011 [4]	Aggarwal and Kehoe, 2011 [4]	Koo et al., 2012 [16]
*N*	432	93	118	70	59	202	20	36	212	119	548 Review^⨀^	272 Review^⧫^	262

Benign						NA						NA	
Teratomas	**39%**	7.5%	**33.9%**	**31.4%**	**42.5%**		15%	17.6%	**35.7%**	**32%**	**34.6%**		**42%**
Cystadenomas	26%	8.6%	29.7%	17.1%	15.3%		25%	8.8%	23.5%	19.4%	23.5%		13%
Endometriomas	13%	2.2%	0.8%	25.7%	5.1%		5%	23.5%	9.4%	3.4%	—		6.5%
Functional cysts^*∗*^	2.6%	**31.2%**	21.2%	20%	15.3%		**30%**	**41.2%**	16.9%	26.9%	—		16.8%
Paraovarian/paratubal cysts	—	3.2%	3.4%	4.3%	—		15%	2.9%	2.8%	13.4%	—		5%
Leiomyomas	—	4.3%	2.5%	—	7%		—		—	2.5%	—		—

Malignant	2.6%	3.3%	6.8%	—	8.5%	NA	10%	5.8%	3.3%	1.7%	5.6%	NA	7,3%
Borderline	**2.6%**	1.1%	**3.4%**	—	1.7%	**56.9%**	5%	**5.8%**	1.4%	—	**3.1%**	**48.1%**	**4.9%**
Epithelial	—	1.1%	0.8%	—	**5.1%**	21.8%	5%	—	0.5%	1.7%	1.6%	21.6%	1.5%
Germ cell	—	1.1%	0.8%	—	1.7%	16.9%	—	—	1.4%	—	0.9%	24.6%	0.8%
Stromal	—	—	1.7%	—	—	0.5%	—	—	—	—	—	NE	—

^*∗*^Functional cysts include follicular formations, *corpora lutea,* and theca lutein cysts.  ^⨀^Review of studies which reported adnexal masses detected and followed up in pregnancy up to the point of definite management (includes Bernhard et al. (1999) [9], Schmeler et al. (2005) [10], Balci et al. (2008) [3], and Yen et al. (2009) [15]). ^⧫^Review of studies including only surgically managed malignant tumors (includes Leiserowitz et al. (2006) [17]). NA: not applicable to study design. NE: not specified.

**Table 4:** Tumor markers in pregnancy [21–27].

Tumor markers	Associated histological types of ovarian and other tumors	Changes in pregnancy	Observations
CA 125	Ovarian epithelial tumors	Increased in 1st trimester: (i) Starts at 30–40 d after LMP, peaks at 35–60 d (ii) May reach 1250 U/mL (iii) Decrease at the end of 1st T	Useful as tumor marker for ovarian epithelial tumors between 15 weeks of gestation and delivery

CEA	Epithelial tumors (particularly, colorectal carcinoma)	Serum levels not influenced by pregnancy	Can be used as tumor marker in pregnancy

CA 19.9	Gastrointestinal, pancreatic, and other adenocarcinomas Ovarian mucinous tumors	Mildly increased levels with increased gestational age, but never exceeding the normal range	Can be used as tumor marker in pregnancy

b-HCG	Germ cell tumors (particularly, choriocarcinoma)	Physiologically increased during pregnancy	Not possible to use as tumor marker during pregnancy

AFP	Germ cell tumors (endodermal sinus tumor, embryonal carcinoma, and mixed tumors)	Physiologically increased during pregnancy Abnormally increased in NTD and decreased in Down syndrome	Serum levels usually <500 ng/mL in pregnancy complicated by NTD and >1000 ng/mL in germ cell tumors

LDH	Dysgerminomas	Increased in pregnancy diseases (severe preeclampsia, HELLP syndrome)	

Inhibin A	Granulosa cell tumors; mucinous carcinoma	Increased in 1st trimester (produced by developing placenta) Abnormally increased in Down syndrome	(i) Used in 2nd trimester for Down syndrome screening (ii) Increased levels require fetal and ovarian evaluation

He4	Serous, endometrioid, and clear cell epithelial tumors	Lower levels in pregnant women Mildly increased in 3rd T compared to 2nd T Elevated levels can also be found in PTD	Promising tumor marker, but its value in pregnancy is not established

NTD: neural tube defects; AFP: alfa-fetoprotein; LMP: last menstrual period; T: trimester; d: day; PTD: preterm delivery.

**Table 5:** Frequency of complications of adnexal masses in pregnancy.

Complications	Frequency
Torsion [2, 3, 5, 9–11, 14, 15, 20]	1–27.7%
Rupture [11, 16]	0–5%
Malignancy [3, 7, 9–16]^*∗*^	0–10%
Abortion [3–5, 10, 14, 28–31]	0–6%
Preterm delivery [3–5]	5.8–10.4%

^*∗*^Malignancy, including *borderline* tumors.

**Table 6:** Size of the adnexal mass and risk of malignancy.

	*N*	Cut-off	Estimated risk
Sherard III et al., 2003 [5]	60	<6 cm	Average lesion size: benign 7.6 cm versus malign 11.5 cm (*p* < 0.05); all masses <6 cm were benign
Schmeler et al., 2005 [10] (masses > 5 cm)	59	—	Average lesion size: benign 7.3 cm versus malign 10,2 cm (*p* = 0.29)
Schwartz et al., 2009 [6]	Review	>7 cm	Refer to only increased risk of malignancy
Yen et al., 2009 [15]	212	≥10 cm	OR: 11.2; *p* < 0.01
Koo et al., 2011 [2]	470	>15 cm	OR: 12.36; *p* = 0.001
Hoover and Jenkins, 2011 [32]	Review	≥5 cm	Refer to only increased risk of malignancy

**Table 7:** Obstetric impact of adnexal masses in pregnancy.

	*N*	Abort.	PTD	Observations
Platek et al., 1995 [31]	31	3.2%	—	59% antenatal surgery
Bromley and Benacerraf, 1997 [28]	125	0%	—	19% antenatal surgery
Whitecar et al., 1999 [14]	130	1.5%	—	66% antenatal surgery
Usui et al., 2000 [29]	69	3.3%	—	99% antenatal surgery
Sherard III et al., 2003 [5]	60	4.7%	9%	100% antenatal surgery
Schmeler et al., 2005 [10]	59	0%	—	29% antenatal surgery
Balci et al., 2008 [3]	36	2.9%	5.8%	94% antenatal/peripartum surgery
Aggarwal and Kehoe, 2011 (review)^⨀^ [4]	498	6%	10.4%	100% antenatal/peripartum surgery

Abort.: abortion; PTD: preterm delivery.  ^⨀^Review of studies including only surgically managed cases of both benign and malignant tumors (includes Whitecar et al. (1999) [14] and Sherard III et al. (2003) [5]).
